# Digital technologies for step counting: between promises of reliability and risks of reductionism

**DOI:** 10.3389/fdgth.2023.1330189

**Published:** 2023-12-13

**Authors:** Alessandra Angelucci, Stefano Canali, Andrea Aliverti

**Affiliations:** Dipartimento di Elettronica, Informazione e Bioingegneria, Politecnico di Milano, Milan, Italy

**Keywords:** step counting, wearable devices, pedometers, reductionism, digital health

## Abstract

Step counting is among the fundamental features of wearable technology, as it grounds several uses of wearables in biomedical research and clinical care, is at the center of emerging public health interventions and recommendations, and is gaining increasing scientific and political importance. This paper provides a perspective of step counting in wearable technology, identifying some limitations to the ways in which wearable technology measures steps and indicating caution in current uses of step counting as a proxy for physical activity. Based on an overview of the current state of the art of technologies and approaches to step counting in digital wearable technologies, we discuss limitations that are methodological as well as epistemic and ethical—limitations to the use of step counting as a basis to build scientific knowledge on physical activity (epistemic limitations) as well as limitations to the accessibility and representativity of these tools (ethical limitations). As such, using step counting as a proxy for physical activity should be considered a form of reductionism. This is not *per se* problematic, but there is a need for critical appreciation and awareness of the limitations of reductionistic approaches. Perspective research should focus on holistic approaches for better representation of physical activity levels and inclusivity of different user populations.

## 1. Introduction

Step counting is among the most basic and fundamental features of wearable technology. Several of the current and very significant functions that wearable devices, also known as wearables, can serve for biomedical research and clinical care are based on the counting of steps to measure and assess physical activity, including mobility, frailty, proactiveness, etc. (1).

The counting of steps that individuals take in specific and significant points or throughout their lives is considered a crucial way of studying physical activity in contemporary societies and its role for individual and population health. In this framework, wearable technologies can be a key resource to develop knowledge on these issues. For instance, wearables are starting to be used to count steps as a way of estimating physical activity in specific and sometimes critical moments, such as before surgeries to estimate clinical risk of individual patients (2, 3). As aggregated data, the abilities of wearable devices to continuously estimate the number of performed steps is also used in large cohort studies that follow individuals for long periods of time to identify correlations between changes in physical activity and health and disease (4).

At the same time, step counting through digital health technologies such as wearables is increasingly at the center of emerging public health interventions and recommendations. For example, the advice of taking a specific number of steps per day (such as the famous “10,000 steps” per day goal) is seen as a crucial strategy to increase physical activity among adults. Wearables and other digital health technologies can and already play a crucial role for these strategies, serving as a tool for monitoring and measuring the number of steps that individual take during a day as well as a way of reminding and nudging individuals to take more steps and reach goals related to specific number of steps (5).

Step counting through wearable devices is thus gaining increasing scientific and political importance in current biomedical research and clinical care. In this paper we provide a perspective of step counting in wearable technology, identifying some limitations to the ways in which wearable technology counts steps and indicating caution in current uses of step counting as a proxy for physical activity through digital health technology. Starting with a review of the current state of the art of technologies and approaches to step counting in digital wearable technologies (Section 2), we discuss limitations (Section 3) that are methodological as well as epistemic and ethical, in the sense that they are limitations to the use of step counting as a basis to build scientific knowledge on physical activity (in other words, knowledge-related and thus epistemic limitations) as well as limitations to the accessibility and representativity of these tools (and thus ethical limitations). In particular, using step counting as a proxy for physical activity is a form of reductionism (6). Whilst this is not *per se* problematic and indeed reductionistic approaches are crucial in the sciences and engineering, reductionistic approaches to physical activity have several limitations and more awareness is needed.

The results of this paper are based on interdisciplinary work, which combines expertise in biomedical engineering and wearable technology development and validation with expertise in philosophy of science and technology and scholars working in in the same department and laboratory.

## 2. Technologies for step counting

Step counting devices, also known as pedometers or activity trackers, have gained significant popularity in recent years, as small, wearable devices have become an integral part of many individuals' daily lives, providing them with valuable insights into their physical activity levels. Wearables exist for different parts of the body. Examples of wearables include watches (such as smartwatches and activity trackers) (3), smart glasses (7), visors (8), rings (9), patches (10), sensorized garments (11), elastic bands applied on the torso (12), earbuds (13), and ankle-worn straps.

Wearables embed different types of sensors, the most common of which are Inertial measurement units (IMUs), barometers, and GPS systems when they are used for step counting. IMUs are commonly used to perform activity recognition, and they can be composed of accelerometers, gyroscopes, magnetometers, or a combination of those sensors. Data from a three-axial accelerometer can be used independently to perform activity recognition and posture detection (12, 14). Miniaturized accelerometers are able not only to detect activities like walking and running, but also to detect when a step is performed from the acceleration patterns (15). Recent studies highlight that the performance of algorithms improves with sensor fusion. In several studies, researchers combined accelerometers with gyroscopes to perform fall detection, a more detailed gait analysis, and gesture recognition. In most circumstances, the accelerometer acts as the lead sensor while the gyroscope functions as the supplementary sensor. There are walking patterns, such as walking upstairs and downstairs, where performance improves when gyroscopic data are used due to the oscillation of the body while climbing stairs (16). Also barometers can be useful in detecting steps while walking upstairs and downstairs due to the differences in elevation detectable from atmospheric pressures (17). Additionally, GPS data can be fused with other sensor data to estimate the number of steps taken, distance and speed. Another interesting opportunity comes from ankle force myography (FMG): since accelerometer-based step counters encounter difficulties in detecting low-speed steps (<0.6 m/s), FMG has been proposed as a solution to count steps (18). FMG is recorded by using force sensing resistors (FSRs) surrounding the ankle to register the volumetric changes during muscle activities. Even during a low-speed walking, the contraction and relaxation of the extensors and flexors different force distributions on the ankle-worn band, resulting in distinctive FMG patterns, and thus allowing to count the number of steps.

By measuring the number of steps taken, step counting devices offer numerous benefits and have revolutionized the way people approach their fitness goals. There are several commercially available devices dedicated to step counting. Some authors propose metrics that combine the number of steps with other measurements, for instance Mishra et al. (19) have defined the index Heart Rate Over Steps (HROS) to detect anomalies in the heart's behavior in relation to the level of activity in pre-symptomatic COVID-19 patients.

An alternative to dedicated devices consists in using smartphones, since they embed the needed sensors to perform the measurements and allow to only install a step counting app. Users can exploit one of the many available step counting apps, or apps embedded in their systems and start tracking their steps daily*.* The Apple Health app available on iPhone, for instance, not only allows to count steps but also estimates other gait parameters (20). Some apps can be paired with other wearables, while other apps can function independently, generally by retrieving data from apps like Google Fit or Apple Health, and provide step counting-related features. There are even some apps which allow to convert the number of steps into a digital currency to obtain discounts, or even cashback like in the case of the app WeWard (WeWard SAS, Paris, France) (21). Limitations of smartphone-based step counting algorithms have been described in the literature in the past few years, such as the different locations where smartphones can be put even by the same user at different times (e.g., trouser pocket and bags), and the influence of non-ambulatory activities and upper arm movements (22). However, the interest in developing such technologies is undoubtedly very high and algorithms for step counting using smartphone sensor data are becoming better and better. Still, smartphones inherently suffer from the same limitation as IMU-based step counters, i.e., low-speed steps are difficult to detect with inertial sensors.

## 3. Opportunities and limitations

An overview of opportunities and limitations of wearables for step counting is presented in Table 1 and discussed afterwards.

**Table 1:** Overview of opportunities and limitations presented by step counting devices.

Type (opportunity or limitation)	Description	Wearables/apps taking advantage of opportunities or overcoming limitation
Opportunity	Nudge to do more physical activity and have a healthier lifestyle	All wearables and apps for step counting, but impact depends on how the solution is designed
Opportunity	Sense of community	Wearables that are integrated with apps with social network features, or apps with social network features
Opportunity	Possibility to implement collective health and wellness programs	Wearables and apps that track more users and allow to visualize aggregated data
Limitation	Sense of pervasiveness	All wearables and apps, but mostly if the users perceive it is not their choice whether to use them (e.g., corporate programs)
Limitation	Lack of accuracy and transparency of step counting	Devices and apps which provide validation data and share them with the users, stating the limits of validity
Limitation	Lack of a comprehensive overview of physical fitness and activity	Sensor fusion from multiple devices and use of several indices, depending on the end user's needs
Limitation	Solution is not inclusive for people with lower-limb impairments	Activity recognition also based on upper-body movements

One advantage of step counting devices or apps is their ability to nudge individuals to lead more active lifestyles. By tracking their progress over time, users are motivated to walk more and have positive physical activity trends. This allows individuals to make informed decision to improve their overall health and well-being, also combining the step counting with other functions such as calories expenditure. For instance, individuals can decide to climb stairs instead of taking an elevator, also because they know that their step count is going to increase.

Step counting apps also offer the opportunity to have a sense of community. Many apps allow users to connect with other people as in a social network, creating a virtual fitness community.

Furthermore, step counting devices have found several applications not only at the personal level but also at the community level. In corporate wellness programs (23), employers have started promoting use of apps and devices to promote an active lifestyle among employees. By encouraging employees to engage in physical activity and to maintain a better lifestyle, companies have witnessed positive impacts on morale, productivity, and overall health and wellbeing. However, monitoring corporate employees poses several ethical and regulatory issues, also tied with the extensive use of artificial intelligence in signal processing algorithms and its inherent lack of interpretability and transparency (24). In the context of step counting and wearable technologies, these issues can lead to the feeling of pervasiveness, stress, and anxiety in users, which in turn pose significant limitations to the extensive use of these devices in the general population. In particular, a lack of accuracy and transparency in step counting has been perceived as a reason for data doubt and anxiety in patients tracking their post-surgery health through commercial wearables, leading some to give up the use of the device (25). In addition, the extensive use of monitoring and data collection allowed by digital health technology such as wearables can be seen as surveillance and monitoring in a context where it is often untransparent and unclear who is responsible for and has access to data on physical activity and how these can be triangulated with other evidence to infer additional information such as location, life patterns, and habits of individual users (26, 27).

Wearable devices can also be applied in the medical field. For instance, it has been demonstrated that the number of daily steps taken by a patient is correlated with clinical metrics of functional capacity, such as the result of the 6-Minute Walk Test (6MWT) (2).

However, in the medical field, step counting has several limitations that prevent it from being used more extensively at present. Factors such as sensor and algorithm accuracy, device placement on the body (including where a smartphone is placed), and individual user behavior can impact the correctness and completeness of measurement. Physical activity guidelines recommend a moderate level of exercise at all ages, and define threshold values for steps walked (28), among other parameters. An example is the threshold of 10,000 steps per day which is often considered as a threshold for a healthy lifestyle according to the recommendation by the Centers for Disease Control and Prevention (CDC) (29). This threshold has been largely put in discussion as it appears arbitrary and does not consider the difference between devices and algorithms. Despite the known benefits of walking for public health, current European guidelines for physical activity have not yet released specific recommendation on the optimal number of steps per day needed for good health and longevity (30). For instance, in the previously cited study on functional capacity (2), the maximum number of daily steps achieved in 7 days of monitoring demonstrated, i.e., the best performance of the week, to be more representative of a good 6MWT results (>350 m) than the mean of daily steps, which was below 10,000 steps for many participants. The 10,000 steps per day (on average) threshold demonstrated its capability to discriminate between clinical scales results, however it is likely that a lower threshold would be sufficient (3).

Additionally, only relying on step counts may lead to overlooking other aspects of physical fitness, such as functional training or flexibility exercises. There is an issue of representation and monitoring of different physical activities when steps are not involved. For instance, cycling is a physical activity but after a session a pedometer, whether embedded in the smartphone or a dedicated device, would not increase its step count. This is a clear example that shows how step counting is not the only possible metric of physical activity. Another limitation that was encountered in previous research work from the authors was on patients with limited lower body mobility such as osteoporosis patients which used step counters as a part of a clinical trial. It emerged that some of their doctors had prescribed the patients several exercises involving the upper body, which causes the step count to remain low even if the patient was in fact doing physical activity. This is also true for wheelchair users, who can be very active in daily life activities and in sports without this being represented by step counting. Finally, the same number of steps can be related to different levels of efforts, such as running on level ground or running uphill. It is essential to view step counting devices and apps as a helpful tool rather than the sole determinant of fitness and activity level.

Regarding algorithm accuracy, a study from 2015 (31) found a strong correlation in step counts between measures of Fitbit and gold standard methods. However, they found low accuracy at slow walking pace, as it was stated in the previous section, which is common in older adults, and pointed out the need for further studies involving the older population, which is the main population when it comes to patient monitoring. There are examples of research efforts to design and validate step counting algorithms based on traditional sensors specifically for slow and intermittent ambulation, such as in the work of Genovese et al. (32), or to find more innovative sensing solutions, like in the previously mentioned ankle-worn FMG (18). This is a crucial aspect of step counting algorithms that should be a focus of future research. In the case of elderly people or people with impairments that cause them to walk slowly, the combination of traditional smartwatches with embedded IMUs and FMG can be of interest, especially because still using a smartwatch would give the person more direct feedback.

Yet, several devices are designed to be sold to amateur or professional athletes, so they are optimized to perform better at faster walking speeds or during running (33). There are for instance specific smartwatch models that are dedicated to specific activities. In general, there is not a one-fits-all solution in terms of sensor fusion: it is important to evaluate one's needs before choosing the right wearable. While commercially available smartwatches can be more than sufficient to monitor most sports activities, there are specific solutions for peculiar sports. For instance, the Garmin Enduro™ 2 (34) and the Apple Watch Ultra 2 (35) are models that are optimized for endurance sports, for instance with a long lasting battery life.

## 4. Discussion

In the previous section, we have identified advantages and limitations involved in the extensive and increasing collection of data on the number of steps walked by members of the general population and their use for building an understanding and assessment of physical activity and health. Identified issues related to the lack of accuracy and significant variability of approaches to measurement can limit the extent to which step counting through wearables can be representative of physical health. As a result, the messages we underscore in this section are that step counting through wearables should not be seen as a necessarily objective and reliable way of estimating physical activity—it should rather be seen as a proxy, a reductionistic way of studying physical health that can create issues of representativity and inclusion.

Our first message is the need to consider and check possible issues of reliability when using wearables for step counting. Step counting through wearables can be misleading and should not lead to thinking that results are necessarily objective and equal to the actual number of steps taken by an individual person throughout their day. There is significant variety in terms of the ways in which sensors collect data and related algorithms estimate the number of steps—to the point that different devices might give significantly different numbers of steps after the same day of measuring. In other words, step counting through digital health technologies is not necessarily a reliable way of getting an accurate estimate of steps. It is neither objective, at least in a simple sense of the notion of objectivity—different tools generate different results based on different assumptions they might make in relation to the ways in which the steps are counted. These are not negative features of the estimation of steps through digital health technologies—but we should not think that this approach to step counting is necessarily reliable and objective.

Rather than thinking that digital step counting is immediately reliable and simply objective, our second message is that the use of step counting to estimate physical activity is a form of reductionism. Reductionism consists in the assumption that some properties or phenomena can be explained, measured, deduced by other properties or phenomena at a simpler or lower level. As such step counting is a form of reductionism because steps are used to measure phenomena at a higher level of complexity and organization, in this case physical activity and fitness. Reductionism is not problematic *per se* and is indeed a standard epistemic strategy that is constantly employed in scientific research and in the clinical practice. For instance, when a case of sickness is explained based on an understanding of specific molecular interactions in the body, the case of sickness is at least partially reduced to molecular interactions. Reductionism is constantly employed in the context of digital health, for example when stress is measured and managed on the basis of physiological signals detected by wearable devices (36).

Yet reductionism can be problematic when the properties, phenomena, measurements at the lower and simpler level are taken to completely explain higher-order phenomena—for instance, when the reduction in measurement is forgotten. The extensive use of wearables and digital step counting and their perceived accuracy runs this risk, and ignoring that steps are a proxy for physical activity can be problematic. For example, the same number of steps might represent very different scenarios in terms of physical activity: the steps might be result of differently intensive physical activities, for instance running or hiking over standard walking, or a combination of all of these. As a result, step counting on its own is not very informative and representative of these aspects of physical activity, and neither can tell us much about the level of activity and functionality involved in a specific number of steps—a particularly significant concern when using digital health technology to count the steps of patients or in clinical settings. We should thus not overlook the fact that steps are not a direct representation of physical activity of physical health, as they are rather a reduction of these phenomena to processes that can be tracked more easily and directly. The specific and reductive ways in which physical activity can be represented based on step counting can also create issues of inclusivity, as individuals with lower-limb impairments and disabilities can be extremely active physically and yet their activity levels might not be represented properly through step counting. These are not completely negative features or inherent limitations of step counting through digital health technologies, but we should not rely acritically on steps as a direct way of evaluating physical activity and health, thus forgetting their reductive and limited features.

Therefore, considering these results and messages, which perspective emerges for future research using or developing wearables for physical activity?

First, when using wearable and other digital health technologies it is crucial to consider the overall goal of their application, which can vary from clinical research to personal fitness, all the way to public health mandates. Accuracy of measurement is in this sense crucial, as we have seen, but should not catalyze all work in the field. Here, wearable device's ability to nudge people into being more active is not necessarily related to accuracy: devices can track trends and encourage positive behaviors. In this sense, a trade-off between high engagement, which can be more easily achieved with contained costs of devices, and accuracy, which requires extensive validation studies in different conditions and thus elevated costs of development, must be found. However, when a specific number of steps is given as medical prescription, for example the previously mentioned 10,000 steps per day, accuracy becomes significant to assess patient compliance.

Second, perspective developments for future research should look towards the integration of several measures in combined and synthetic indices and the recognition of activities based on multiple parameters. Consider for instance combined indices that are already used in cardiovascular research, such as the previously defined HROS for measuring the response of the cardiovascular system during lower limb physical activities (e.g., walking, running, climbing stairs) (19): this index is more informative than heart rate, which does not consider effort levels and physical activities. Several wearables already employ artificial intelligence and machine learning algorithms to recognize activities—this direction can help expand the limitations of step counting and move research toward a more holistic consideration of different types of physical activities. These are very interesting and promising directions, but reductionism might still negatively limit their success and there might be additional concerns on the accessibility and explainability of combined indices and activity recognition.

## 5. Conclusion

The increasing availability and use of wearable technology by the general population is leading to a variety of applications as tools for biomedical research and clinical care. In this paper, we have discussed a specific yet very significant and prevalent use of wearables—step counting as a way of estimating and representing physical activity and health. While we see great potential in this direction, based on a review of current technologies and approaches we have cautioned against current limitation of using steps as proxies, because of the variability of measurement of steps by wearable technology and the constraints of steps as an index of physical health. We have argued that this use of wearables is a form of epistemic reductionism, whose limitations need to be front and center in current health campaigns based on digital therapeutics and research studies based on digital health. Perspective research should focus on holistic approaches for better representation of physical activity levels and inclusivity of different user populations.

## Data Availability

The original contributions presented in the study are included in the article/Supplementary Material, further inquiries can be directed to the corresponding author.
